# Traditional Arabic & Islamic medicine: validation and empirical assessment of a conceptual model in Qatar

**DOI:** 10.1186/s12906-017-1639-x

**Published:** 2017-03-14

**Authors:** Sara N. AlRawi, Amal Khidir, Maha S. Elnashar, Huda A. Abdelrahim, Amal K. Killawi, Maya M. Hammoud, Michael D. Fetters

**Affiliations:** 1 Department of Family Medicine, University of Michigan, 1018 Fuller Street, Ann Arbor, MI 48104-1213 USA; 2 Weill Cornell Medical College in Qatar, Education City, PO Box 24144, Doha, Qatar; 3 Department of Obstetrics and Gynecology, University of Michigan, 1500 E. Medical Center Drive, L4000 Women’s, Ann Arbor, MI 48109 USA

**Keywords:** Traditional medicine, Prophetic medicine, Graeco Arabic medicine, Unani medicine, Quranic healing

## Abstract

**Background:**

Evidence indicates traditional medicine is no longer only used for the healthcare of the poor, its prevalence is also increasing in countries where allopathic medicine is predominant in the healthcare system. While these healing practices have been utilized for thousands of years in the Arabian Gulf, only recently has a theoretical model been developed illustrating the linkages and components of such practices articulated as Traditional Arabic & Islamic Medicine (TAIM). Despite previous theoretical work presenting development of the TAIM model, empirical support has been lacking. The objective of this research is to provide empirical support for the TAIM model and illustrate real world applicability.

**Methods:**

Using an ethnographic approach, we recruited 84 individuals (43 women and 41 men) who were speakers of one of four common languages in Qatar; Arabic, English, Hindi, and Urdu, Through in-depth interviews, we sought confirming and disconfirming evidence of the model components, namely, health practices, beliefs and philosophy to treat, diagnose, and prevent illnesses and/or maintain well-being, as well as patterns of communication about their TAIM practices with their allopathic providers.

**Results:**

Based on our analysis, we find empirical support for all elements of the TAIM model. Participants in this research, visitors to major healthcare centers, mentioned using all elements of the TAIM model: herbal medicines, spiritual therapies, dietary practices, mind-body methods, and manual techniques, applied singularly or in combination. Participants had varying levels of comfort sharing information about TAIM practices with allopathic practitioners.

**Conclusions:**

These findings confirm an empirical basis for the elements of the TAIM model. Three elements, namely, spiritual healing, herbal medicine, and dietary practices, were most commonly found. Future research should examine the prevalence of TAIM element use, how it differs among various populations, and its impact on health.

**Electronic supplementary material:**

The online version of this article (doi:10.1186/s12906-017-1639-x) contains supplementary material, which is available to authorized users.

## Background

Global use of traditional medicine has continued to gain momentum over the last decade [1]. Evidence indicates traditional medicine is no longer only used for the healthcare of the poor, its prevalence is also increasing in countries where allopathic medicine is predominant in the healthcare system [1]. The World Health Organization (WHO) defines traditional medicine as “the sum total of the knowledge, skills, and practices based on the theories, beliefs, and experiences indigenous to different cultures, whether explicable or not, used in the maintenance of health as well as in the prevention, diagnosis, improvement or treatment of physical and mental illness. Traditional medicine practices vary greatly from country to country and region to region, as they are influenced by factors such as culture, history, personal attitudes, and philosophy” [1]. Traditional practices are based on a holistic approach to the human being within the wider environment; it is a framework that reaches far beyond the field of health to the broader level of society, religion, and culture [2].

It is imperative to note that the terms complementary and alternative medicine refer to “a broad set of health care practices that are not part of the country’s own tradition and are not integrated into the dominant country's health care system” [1]. In some countries, these terms, along with non-conventional medicine, are at times used interchangeably with traditional medicine [1]. For the purposes of this research, we have chosen an emphasis on traditional medicine.

Previous research illustrates the traditional medicine usage patterns in a variety of countries. Over 80% of the world population depends on herbal medicines [3], and current estimate of herbal medicines’ global market have surpassed $60 billion USD [4]. The WHO, China, India, Nigeria and the United States of America (USA) have invested considerably in traditional herbal medicines research, a significant step towards global health [5]. In 2010, Qatar’s pharmaceutical market was estimated at QR 1.43 billion ($392.6 million USD) [6] with research illustrating a high demand for herbal and nutritional supplements [7]. Complementary and alternative medicine prevalence in some developed countries is between 70% and 80% [4]. Countries throughout the Middle East including Egypt, Jordan and the Arabian Gulf have began incorporating herbal CAM along with conventional medicine into the National Health Services [8]. Major challenges exist in the implementation of traditional, complementary, alternative and herbal medicine as it pertains to regulatory status, efficacy, quality control and safety monitoring [9].

A prominent traditional healing system in the world, Traditional Arabic & Islamic Medicine (TAIM), refers to healing practices, beliefs, and philosophy incorporating herbal medicines, spiritual therapies, dietary practices, mind-body practices, and manual techniques, applied singularly or in combination to treat, diagnose, and prevent illnesses and/or maintain well-being [10]. Despite remarkable advancements in orthodox medicine, traditional medicine has been practiced in the Middle East since ancient times. For those dealing with ailments such as infertility, psychosomatic troubles and depression, TAIM is often the first choice of treatments [7]. Traditional Arabic Medicine is the culmination of Graeco-Roman, Chinese, Persian, and Ayurvedic theories and practices and continues to be practiced in parallel with modern, orthodox medicine [11]. Origins of Islamic medicine can be traced back to the beginning of the Islamic civilization in the 7th century when Islamic scholars and physicians expanded earlier medical sciences with their own discoveries [11], and amplified preexisting theoretical principles of medicine into a comprehensive system of medicine [12]. Within this framework, there is a humoural and temperamental etiology to disease, a spiritual influence according to Abrahamic scripture, and the healing power of nature is utilized for health restoration and preservation [12].

TAIM reflects an enduring interconnectivity between Islamic medical and Prophetic influences, as well as regional healing practices emerging from specific geographical and cultural origins [10]. This amalgam of indigenous medical knowledge can be found in current ethno botanical surveys, which document the use of herbs for cancer care in the regions of Israel, Syria, and the Palestinian Authority [13]. Use of traditional medicine, particularly herbal medicine, can further be seen throughout the Middle East. Reports indicate 200–250 plant species are still in use in traditional Arab medicine for the treatment of various diseases [14], where many of the herbs are used in a culinary and medicinal manner. This is illustrated by the use in foods of biologically active compounds such as Taraxacum, Black Cumin, Chaste tree, Chicory, Snakeroot, and French Lavender [15].

Despite the previous development of a theoretical TAIM model that unites inter-related and overlapping terminology, empirical support for the model to actual use and motivations for use have yet to be demonstrated. Moreover, little is known about how patients discuss TAIM use with allopathic medicine providers. Hence, the primary objectives in this research are: 1) to provide empirical support for the TAIM conceptual model and illustrate its real world applicability by examining patients’ reports on their use and reasons for using TAIM, and 2) to understand how patient-doctor dynamics impact disclosure of TAIM. A parent investigation conducted in Qatar about comprehensively assessing healthcare quality described previously [16], provided a large qualitative database to investigate patients’ experience with both conventional and alternative healing practices.

## Methods

We used an ethnographic approach to conduct a qualitative study as one component of a multistage, mixed methods parent study focused on developing a self-administered healthcare quality assessment instrument in the four languages of Arabic, English, Hindi, and Urdu [17]. We used Kleinman’s conceptual model, “Cultural Construction of Clinical Reality” to inform the study [18]. The initial stage of the parent study involved cultural adaptation of the Consumer Assessment of Healthcare Providers and Systems Survey [16]. The second stage of the parent study involved qualitative interviews regarding patient perspectives on healthcare quality, including the use of alternative medical practices.

### Setting

Qatar has a population of approximately 1.9 million people [19], with the majority comprised of expatriate workers from all over the world temporarily residing in Qatar, and a minority of Qataris [19–21]. Doha presents an extremely high-density multicultural setting [22]. Outpatient clinics in the Hamad Medical Center, the leading healthcare provider in Qatar, served as the specific data collection site.

### Sampling and enrollment procedures

To attain a minimum of 20 participants per language group for in-depth interviews to ensure data saturation, we targeted the recruitment of approximately 80 subjects. Female research assistants, who can move between the gender segregated waiting rooms, approached patients and/or family members in the outpatient waiting room of the clinic to recruit them. The inclusion criteria were: 1) Speaks as first language the target language – primary language was defined as a language that the participant grew up speaking and/or reading from his or her childhood, or as determined by sociocultural norms, such as work environment of his or her home country 2) Has lived in Qatar for at least 12 months in the past 3 years 3) Provides verbal informed consent. Exclusion criterion was 1) Has a severe debilitating illness precluding participating.

### Data collection

Items on the semi-structured interview guide for the parent study on developing a culturally adapted survey on healthcare quality ascertained an individual’s health and illness experiences, health observances amongst other healthcare practices, challenges encountered when receiving healthcare, recommendations for improvement in services, as well as demographic information.

The interviews were conducted by research assistant-(RA) pairs, each bilingual in Arabic and English, Hindi and English or Urdu and English, who respectively conducted the Arabic, Hindi and Urdu interviews. The English interviews were conducted by various combinations of RAs depending on recruitment and RA availability. The senior PI on the project, MDF, a qualitative and mixed methods research methodologist, trained the interviewers. As the first interviews were completed, these were reviewed to help further refine the training of the RAs, and to begin the iterative analysis. Transcripts were not returned to participants as this was not really feasible given the high mobility of the engaged population, as many are transient workers, or are private and reserved. Field jottings were recorded in the field, and then expanded into full notes after the interviews. The duration of the interviews was 15–60 min. There were no focus groups. The RAs were trained to record demographic information, context, content and conceptual ideas. Observations were debriefed on a regular conference call held approximately every week. A sample of the interview guide can be found in Additional file 1. A full paper based heavily on the field notes as well as interview data about recruitment and informed consent has been previously published [22].

### Data analysis

Audio-recorded interviews were transcribed in the native language, and a second reviewer checked all transcriptions. Any possible identifying information was modified to protect privacy. Arabic, Urdu, and Hindi transcripts were independently translated into English, compared for similarity, and differences resolved by a third bilingual researcher.

To develop preliminary codes for systematic analysis, team members (AK, MF, and a research assistant) immersed themselves in the data by independently reading and open coding transcripts. Codes and definitions were routinely reviewed and refined during meetings. Emergent themes were amalgamated into the coding scheme, and coding definitions were developed through general consensus of the team. Two analysts (AK and the research assistant) independently coded two transcripts and compared for calibration, while a third (MF) reconciled any variations. All of the remaining transcripts were coded by the primary analyst (AK), who also consulted with team members each week to resolve emerging concerns. The qualitative analysis software ATLAS.ti [23] was used.

The major codes, Food/Diet/Drink/Nutrition/Supplements, Religion, and Interpretation of Health & Illness were used to generate an initial summary for review. The search was then refined for output by language and gender. Data collected on the demographics instrument was merged with textual data to provide context for stories and quotes. From the generated outputs, we looked for elements of, and participants’ experiences, with the TAIM conceptual model, including herbal medicine, spiritual healing, dietary practices, mind-body applications, and manual techniques. We looked but found no other traditional healing practices or evidence contradicting the model. Finally, we searched text for examples of participants’ experiences and reflections about discussing their use of these practices with their doctors. We integrated the diverse data sources into a narrative format to address these study purposes.

## Results

### Participant recruitment

A total of 84 individuals (43 women and 41 men) participated and fell into age categories between 18 and 74 years of age, had varying educational backgrounds from no formal education to primary education only, with religious affiliation of mostly Muslim, but also included Christian, Hindu, and other/not reported. For full details on participant recruitment, see another previously published study [24]. Pseudonyms are used rather than participant numbers. The pseudonyms are consistent with the cultural background, thus conveying quotes from a variety of individuals of varied backgrounds.

## Elements of TAIM conceptual model

**Fig. 1:**
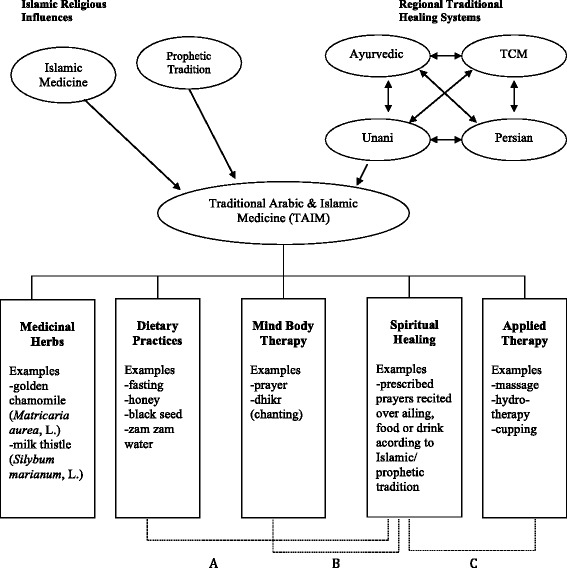
A Unifying Conceptual Model of Traditional Arabic & Islamic Medicine (TAIM), Reproduced with permission from Global Journal of Health Science. A, Dietary practices derived from Islamic/ prophetic tradition include prescription for fasting and drinkingZamzam water. B, Mind-body therapy practices originating from Islamic/ prophetic tradition include prayer. C, Applied therapy consequential of Islamic/ prophetic tradition include cupping

Participants discussed traditional healing practices as attributable to Islamic tradition, namely, practices based on Islamic religious texts and worship practices, as well as those outside the scope of Islamic tradition, stemming from cultural or ethnic heritage unique to a geographical area. As previously proposed, empirically, Islamic worship practices and regional healing practices proved to be at the heart of the TAIM conceptual model (Fig. 1), and can be further classified using the categorization of herbal medicine, spiritual healing, dietary practices, mind-body applications, and manual techniques. For examples of supplementary supportive quotes in the narrative that follows, please see Table 1.

**Table 1:** Supportive quotes for TAIM healing practices

Spiritual Healing
• “In spiritual, they told me to recite verses of the Holy Quran, and I mean, after reciting, blowing on the water and making her drink, to do Dum (reciting the verses and blowing on the person) so I did it. And those pir faqir, (spiritual leaders) they also used to give amulets etc.” • “They also used to recite but they also used to tell me that you read this, such as you blowing on her after reciting Alhumd Surah Alfatihah, (first chapter of the Quran) seventy-two times. I still do it even now but when she gets very ill then I surely read it on her because I see a lot of benefit of it.” • “Only that whenever we take medicine we pray for cure as well. The first we do is to pray for cure when taking any medicine. Religion has the priority.” • “I also got that treatment a lot, they write Quran and Hadith on a clean paper and then they drink it by putting it in the water and take a bath with it, so I also did that.” • “…I’m a good Christian and my believes…you know, I was brought up that you pray to a certain saint.”
Herbal Medicine
• “So these people give the treatments like hakims (religious teacher), they make medicine from herbs.” • “If I find it somewhere, they I pick a few seeds and eat though it is full (of cure). It is said for the black seeds that…there is a Hadith (saying of the Prophet Muhammad), it has cure for everything except death.” • “I do not use toothpaste, I mostly use miswak (a teeth cleaning twig made from salvadora persica tree) according to the Sunnah.”
• “I take a tea called Rooibos tea, which is a South African tea and its not a leaf tea, it’s a bush and it has no tannin and its high on anti-oxidants.” • “There was a pain in abdomen, severe pain, he gave me a medicine made out of roots.” • “Now what I have done is we take Ayurvedic treatment…” • “My brother in law has the knowledge of homeopathy, so I use it sometime when he got to know about my illness, he sent a bottle.”
Applied Therapy
• “It is Sunnah (what Prophet Muhammad [peace be upon him] said or used to do) if someone wants to do (use cupping).” • “Got the treatment from the doctor too but when it didn’t get well then do the massage, then they pull, then it gets well…” • “A man told me that there is a man in Pakistan who blows on people, and later he cuts wood as he cuts the plant, he will blow and will hit with it also, so I thought of getting it done when I will go this time.” • “There is also vapourisation.”
Mind-Body Therapy
• “I meditate.” • “When you do the ablution for the five daily prayers, your sins fall from you like dirt from the body.” • “To stay healthy…just one should pray five times. It means to stay away from bad habits so that one stays healthy.”
Dietary Practices
• “Because you see in religion you are taught that you have to respect your body, so if you respect your body, you have to eat the right food and you should not neglect your body.” • “There is saying of our Prophet (peace be upon him) that while eating leave a little hunger, it is better. Same is for water, if you drink in the beginning then you will eat balanced diet.” • “Religion tells you to eat permissible tings and not to eat not permissible things, and secondly eat according to the way of the Prophet (peace be upon him).” • “You know I look certain food stuffs, things like, you know the traditional food stuffs supposed to give you health, ginger, chili, you know those sort of things…you know, supposed to help garlic.”

*Note*: The statements in Table 1 are not English translations, we sought to reflect the language participants actually used with a high level of fidelity in our representation. It serves the purpose of illustrating the difficulty of what the participants have said. For the non-English language, further, it provides authenticity to the metaphors used which are virtually impossible to find equivalents in English. Also, many of the participants had a low level of literacy, and in fact spoke ungrammatically

Many of our participants spoke about Islamic-based practices. As the Quran and Prophetic traditions or *hadith*, are the textual foundations of the Islamic faith, references to herbs, dietary practices, or other healing practices in the Quran and Prophetic traditions formed the basis of specific healing practices. Islamic religious texts are also the primary source for Islamic worship practices, correspondingly perceived to have healing characteristics. Within Islamic worship practices, a spectrum of therapies related to spiritual healing, herbal medicine, manual techniques, mind-body therapy, and dietary practices can be extrapolated. Our findings reflect the greatest emphasis on spiritual healing, dietary practices, and herbal medicine of the five proposed elements of the model.

### Spiritual healing

Many participants believe that God trusts the individual with good health; God sends down illness and is the One who ultimately relieves it. Muslim participants in particular often described how they use Islamic texts, Holy Quran and Hadith, or Prophetic tradition as primary sources of prayers for healing. These descriptions occurred for both individuals in their approaches for themselves and for treatment of others. For instance, participant Omar states, “if you read translation of the Holy Quran then you will know it is about cure…” This idea is also illustrated by Ali, who said “I had pain here (he pointed towards his chin), then later my father told me to recite Alhumd (first chapter of the Quran) three times, seven times Qulhu walla hu Ahad (chapter 112 of the Quran), and blow, then if God wills then this problem will be gone.” In referencing Hadith, or Prophetic traditions, Mohamed states “there are many Hadith and there are (instructions) in the religion about health.”

Spiritual healing practices are also carried out in the manner of religious figures reciting prayer over those who are sick, or over things consumed by those who are sick. In a similar manner, individuals also recite prayers as a form of self-care. Examples include prayers recited over water. Mariam states, “…those verses of cure, I mean, even the prayer for eye-sight. First, I have to believe in this then doctor…so I used to read the healing prayer of eye-sight and used to blow on Zamzam water (a well in Mecca, believed to be a miraculously generated source of water from God) and I prayed from my heart and applied it on his eye. All praise to Allah, now his eye is fine.” Specific prayers, or supplications, may also be recited aloud or silently by the ailing over the afflicted body area. Noura states, “…it is like, when you pray, you read something and blow on the palms and rub… where there is pain.” Mona states “…every day I read in the morning and blow on the water. I drink it myself and also give it to the children to drink.”

### Herbal medicine

Herbal remedies appear to be mainly used in a culinary manner, and reflect the integration of these practices into the mainstream diet. Nigella sativa (L.), an herbal remedy used by subjects and commonly known as black seed, is referenced in the Prophetic tradition as having healing qualities. Use of black seeds is illustrated by participant Farah who states, “If you use black seeds, probably if you add them in cooking as I do…I must put a little in cooking, with the Sunnah point of view” (Sunnah, things done or permitted by prophet Muhammad [peace be upon him]). A woman, Henna states “I used a little black seeds in food according to the Sunnah, in gravy, I put in everyday food.” Mohamed describes, “what my mother did that she warmed a garlic clove in the oil and as soon as she put, after 2–4 s my pain disappeared.” Sayed explained, “all our life we are using the traditional healing… if someone gets a sun stroke then he gets wrapped with Sin and Itrishia (pelargonium graveolens) and Fliyo (mentha pulegium) just like the rose water and flower water itrishyia (pelargonium graveolens) water, and Fliyo (mentha pulegium) water…”

### Dietary practices

Therapies related to diet are often a form of self-care amongst our participants, and include the use of foods such as honey, also mentioned in both Prophetic tradition as well as the Quran. Ali illustrates use by saying, “if you are taking honey then you will not have any problems.” He goes on to further say, “you know in our Islam, there is so much already; I mean you take proper diet. While eating, drink water before or in the middle not at the end, if you follow these you would be safe from many diseases.” Saif reports, “his way (way of the Prophet Muhammad [peace be upon him]) was to eat while sitting on the floor, it means, should wash hands, start with the name of Allah, and drink water in the beginning, can drink in the middle but it’s better not to drink at the end. The last, it means one should not eat that much, that it puts burden on your abdomen, if it will be (heavy) on your abdomen then it will be on the whole body.” Yet another opinion, Nadia stressed the importance of “drinking Zamzam water.” Faisal discussed the value of dietary practices by saying “traditional food stuff supposed to give you health, ginger, chili, you know those sort of things…supposed to help, garlic…”

### Mind-body therapy

The ritual prayer, salat, incorporates a specific set of physical postures while reciting specific verses of the Holy Quran, glorifications, supplications, and affirmations to God. Our respondents felt that ritual prayer promoted well-being. Notably, Kashef states, “the (effect) of religion is that in our religion, e.g., there is prayer, if you pray regularly then your health will stay good without fail… it is also a kind of exercise. If you pray five times a day then there are so many units of prayer that one gets good exercise.” The Islamic ritual prayer may also be seen as a type of active meditation, where each posture promotes physical and psychological well-being. Saima illustrates this point stating, “praying five times a day is very important both spiritually and physically as well… it’s a matter of composing your body and making the movements for your prayer…sometimes for example if I am feeling stressed if I pray then everything is relieved for me.”

### Applied therapy

Al-hejamah, the Arabic term for cupping, literally means to reduce in size or to return the body to its natural and harmonious state. The practice of al-hejamah has pervaded the Middle East for thousands of years with citations dating back to the time of Hippocrates. Also taken from Prophetic tradition, this is a method whereby blood is drawn by vacuum from a small skin incision for therapeutic purposes. Ibrahim states, “… it is Sunnah (what Prophet Muhammad [peace be upon him] said or used to do) if someone wants to do” (use cupping). Another participant Hussain states, “I thought about doing cupping soon, as it might be useful to liver diseases…” Ayesha spoke about her perception related to the use of cupping, “certainly cupping I know is quite popular.” Mary notes yet another variation, “…Vapourising…inhaling with hot water… during an illness or for preventive measures.”

## Patient doctor dynamics as determinants of TAIM disclosure

An additional key finding highlights the dynamic in which patients interact or don’t interact with their doctors as it relates to disclosure of TAIM use. Participants were more likely to use vitamins and herbal therapy if recommended by their doctors. Sanaa states when asked about use of complementary therapies, “not by myself…only when doctor prescribes…because we are not aware of their side effects.” Omar further supports this notion when asked about using non-medical interventions, “No whatever doctor recommends… they usually prescribe B-Complex.” Saima notes, “Whatever doctor recommends… we take things according to that”.

For non-Qatari’s, relying on their doctor for guidance is instrumental, as they may lack familiarity with non-allopathic care. Ali states, “there is no system of herbs here… when a person is in his own country, then he uses all the treatments.” When asked about her use of herbs, Isra states, “No, didn’t do because I’m here since the age of 19. I came here right after getting married…there is no system of herbal here.”

Participants had varying opinions as to the role their doctor plays in prescribing or informing healthcare choices relating to TAIM. Some participants believed it was the doctor’s responsibility to inquire about any and all matters related to their health and well-being. Ali states, “If doctor would ask then we will tell him that we have taken such treatments,” while Mary also supports this idea, “Doctor should ask about other treatments we are taking.” When asked if her doctor is aware of her intake of foods and drinks to maintain health, Hala notes, “…No the doctor doesn’t know, I mean the doctor didn’t ask me.” Other participants believed use of traditional herbs contrasted from medicine, and if they are perceived as safe, then do not warrant disclosure. Karima states, “herbs and stuff like that is separate, medicinal herbs will relax, these are all beliefs that apply to each individual’s beliefs and customs.” Samiah states, “…natural known herbs… the things that are from our parent’s experiences… since we were young make me to drink it with milk; these are good, I mean they are natural.” Samiah goes on to say that she doesn’t feel its necessary to disclose use of such herbs, “because…there is no harm out of it.”

## Discussion

Intuitively and anecdotally, there has been an understanding that various elements of the TAIM model are utilized in the Arabian Gulf. Supporting the study’s first two specific aims, this is the first research report providing empirical support for the TAIM conceptual model and illustrating its real world applicability evidenced by patients’ reports on their use and reasons for using TAIM. Additionally, this research contributed to our understanding of how patient-doctor dynamics impact disclosure of TAIM. Importantly, there is not an equal distribution of the elements of the TAIM model, with more practices grounded in herbal medicine, spiritual healing, and dietary practices.

Herbal medicine use stemmed from religious texts, as well as traditional practices unique to a geographical area. Herbs were mostly used in a culinary manner, while traditional herbal preparations including tinctures, teas, or salves were not as prevalent. Non-Qatari’s spoke about purchasing herbal remedies upon returning to their homeland, or asking family members to send via mail, suggesting a possible lack of access to herbal formulations and cost may be a barrier if it is an out of pocket expense. Doctors at HMC prescribed multivitamins, prenatal vitamins, as well as individual vitamins. Thus, if doctors prescribe vitamins or nutrients, then patients’ perception may be that additional herbs are unnecessary. Gerber et al. found among midlife Arab women living in Qatar, nutritional remedies and herbal remedies were the most frequently used types of CAM, followed by physical methods [25].

Prayer (salat), Dhikr or remembrance of Allah, and recitation of the Quran are common examples of spiritual healing practices employed by our participants. Recited prayers and worn amulets contain verses from the Quran, to which curative powers are ascribed as passages of the Quran are believed to have vast healing properties [26, 27]. For instance, when Surah (or Chapter 38 of the Quran) is recited on a sleeping person, it cures breathing problems; when written down and read during a patient’s waking hours, it cures illness; and when a person continuously recites this chapter he becomes immune from all troubles at night [28]. Once a spiritual prescription has been given for a particular condition, it can be used continuously over time until the ailment is resolved, thus the need for repeated visits may be unnecessary.

Participants spoke about dietary practices that derived from both geographical influences, as well as religious texts. Examples of geographical influences include the use of spices, such as ginger and chili, in a culinary manner to aid health and well-being [29]. The Holy Quran and Prophetic tradition provide guidelines on the manners of eating and drinking, as well as etiquette related to before, during, and after finishing eating [30]. Making supplication before and after each meal, eating with the right hand, eating seated, slowly, and in moderation are some examples [30]. Eating is considered first an act of worship and second for maintaining good health [30].

Many religions and spiritual traditions across the world ascribe beliefs in healing through prayer. The Islamic ritual prayer (salat) involves certain physical postures and can be comparable to active meditation. Prayer is a special form of meditation and may therefore convey all the health benefits that have been associated with meditation [31] including enhancing spiritual, psychological, and physical well-being. Our participants did not ascribe prayer to being a mind-body practice, and most would consider it first and foremost an act of worship. For this reason, we grouped all references to prayer and divine remembrance under spiritual healing.

Applied, physical or manual therapies include any practice whereby physical application occurs, and includes such therapies as reflexology, cupping or hijamah, and bone setting. This element was the least prevalent among our subjects, and also influenced by religious and regional forces. Islamic books state that the prophet Mohammed (peace be upon him) referred to three methods in curing an illness: “*a drink of honey, a scratch of hijamah and cautery.*” [32] Though the Chinese and the Arabs have practiced cupping since antiquity, the application of this method varies from region to region [32]. While some applied therapies can be self-administered, of the proposed elements, this particular one requires access to a trained practitioner and may become a barrier in access to care.

Traditional medicine practices are “woven into everyday life and belief systems”, and traditional healers are “trusted members of the community” [33]. Thus, traditional medicine is the primary source of health care at the community level [33]. Cultural beliefs and practices often lead to self-care or home remedies in remote areas and consultation with traditional healers [14].

This research also elucidates the details and motives that affect use of TAIM in this population. Muslim participants are motivated to use traditional practices, as they are believed to stem from religious texts, and consequently for many, an extension of worship practices. Amongst non-Muslim participants, worship practices such as prayer are also believed to have curative properties. Moreover, participants relied on traditional medicine if access to allopathic doctors was limited. This is evident in remote areas where doctors or specialists may not be available [34]. Traditional practices are also utilized if the issue is believed to be of a psycho-spiritual nature, and thus independent of a medical nature. Truter (2007) [35] ascribes patient reasons for visits to traditional healers as those not only for health concerns but also for illness stemming from super- natural causes, as well as a lack of trust in the ability of Western medical practitioners to effectively treat psychosocial problems.

Participants spoke of utilizing traditional therapies while visiting their home country; some were not familiar with whether or not an herbal system exists in Qatar and relied on family to supply them with needed herbs or homeopathic formulations. Based on these participants’ conversations, they entrust traditional healing practices as they are deemed in line with their cultural and philosophical belief system, and thus a primary choice for maintaining health and wellness. In one study, the authors report that almost half of the respondents interviewed have used herbal supplements, vitamins and minerals, as well as non-vitamin, non-mineral, and non-herbal supplements, and preferred herbal supplements to conventional medicine for addressing digestive ailments, common respiratory concerns, and weight support [36].

In consideration of our final objective, this research highlights the dynamic in which patients interact or do not interact with their doctors, as it relates to disclosure of TAIM use. Participants were more likely to use traditional therapies if recommended by their doctors, and participants also had varying opinions as to the role their doctor plays in prescribing or informing healthcare choices. Thus, education, life experiences and the economics of a community may impart whether an individual may or may not initiate healthcare seeking behavior [37]. Self-reported use of CAM among the healthcare consumers at a tertiary care center in UAE demonstrates that 70% of the users did not consult any physician, but used it with non-medical information [8]. A review of qualitative and quantitative studies conducted in the US between 1993 and 2002 found the rate of non-disclosure of those using CAM is as high as 77%, and includes reasons such as concerns about a negative response by the practitioners, the belief that the practitioner did not need to know about their CAM use, and the fact that the practitioner did not ask [38]. In exploring self-treatment practices originating from popular, folk, and professional sectors, patients most likely discussed treatments from the professional sector with physicians because they were perceived as legitimate and medically acceptable, furthermore suggesting that patients may be more willing to disclose use of provider-based CAM (e.g., chiropractic or acupuncture) relative to self-care CAM (e.g., vitamins and herbal medicine), as the former is perceived as more legitimate [39].

Doctors at HMC prescribed individual vitamins, multivitamins, as well as prenatal vitamins thus the perceived need of supplementing may be deemed ill-advised or unnecessary. A cross sectional study examining the knowledge, attitudes, and practice of general practitioners towards CAM in Qatar determined that over 39% reported poor knowledge about CAM [40]. Additionally, many of the general practitioners interviewed did not refer their patients for CAM or inquire about patients’ use of CAM therapy. Lastly, their lack of knowledge in CAM was seen as a barrier to its use by 60% of the general practitioners interviewed. While Qatar is characterized by a mix of ethnic and cultural groups, there is a need to identify further the types of traditional practices being used, the frequency of use, factors affecting use, patterns of utilization, and most importantly their contribution to health.

### Limitations

Regarding potential limitation of this research, first, while Qatar is demographically diverse, it is unclear to what extent variation can be generalized to other parts of the Middle East. Ethnographic research is best suited for demonstrating patterns and meanings of use, rather than assessing prevalence of use. This would be an area ripe for future investigation. That said, there are several other countries that rely heavily on expatriate workers, e.g., UAE, Bahrain, and Kuwait where these findings may also apply. To examine fully the prevalence and utilization patterns of TAIM, the future studies should include both private and government healthcare facilities. There is open opportunity for direct research in settings where all forms of TAIM are provided. Second, there is the potential for desirability bias. In this case, the normative basis would be for participants to praise the system, and indeed we found some evidence of individuals who said so. As our interviews asked individuals about their experiences, and not their attitudes, this concern, we believe has minimal impact. Lastly, we were aware of our bias going into the data set, and we looked for disconfirming information, and also evidence for other elements, but these were not identified. All types of treatments that were discussed fall under one of the TAIM components.

## Conclusion

Based on our findings, we conclude that the TAIM model is theoretically robust, and has an empirical basis for all of the elements in the TAIM conceptual model. These findings further suggest that orthodox medicine practitioners will need to inquire if they want to learn about their patients’ use of TAIM elements. Traditional medicine provides a platform for ensuring that all people have access to care. As traditional medicine is either the foundation of healthcare delivery, or serves as a complement to it, we recommend further exploration into traditional healing practices.

## Additional file

Additional file 1: Sample Interview Guide. (DOC 40 kb)

## Abbreviations

CAM: Complementary & alternative medicine
TAIM: Traditional Arabic & Islamic Medicine
WHO: World Health Organzation
