# Supplementary material for: Traditional Arabic & Islamic medicine: validation and empirical assessment of a conceptual model in Qatar
Source: BMC Complement Altern Med. 2017 Mar 14;17:157. doi: 10.1186/s12906-017-1639-x (PMC5351166; doi:10.1186/s12906-017-1639-x)
Supplement: Additional file 1: — Sample Interview Guide. (DOC 40 kb) [file 12906_2017_1639_MOESM1_ESM.doc]

Appendix A

***Interview Guide***

| **Key Interview Points**   - **What is best term for primary care doctor? Their clinic?** - **To what extent do patients feel family should be a part of their care when ill?** - **What do patients know of supplemental health insurance?** - **How does gender matter in doctor-patient relationships?** - **How should doctors address the religious influences on patients?** - **What information should doctors know about traditional healing practices, eg, food, drink, herbs, other treatments?** |
| --- |

**Introduction**

I am one person on a research team looking at how to improve health care quality. I would like to discuss your opinions about this. Your opinions are VERY valuable as we try to learn how to improve the quality of medical care for you and your community. I would like to tape record our conversation so we can be certain not to lose any information you tell me. After the recording is reviewed to check my notes, the recording will be deleted.

Transition: We are interested in learning about what happens when visit your doctor.

Could you think back to a recent illness when you saw the doctor.

**PROFESSIONAL DOMAIN**

**Family and Doctor Relations**

1. What made you realize you are ill? What were you experiencing when you first felt ill? .
2. When you got ill who did you turn to for advice about what to do?
3. What do you usually do?
   1. Probe: what role does your family play when you are ill?
4. How did you decide when to go to see a doctor?
5. What do you usually do?
6. Are there things you look for when choosing a doctor?

Probes: language, gender, religious concordance

1. We are developing a survey for many people and want to find the best way to refer to the doctor that you usually see when you first feel ill. What do you call this doctor you see when first ill?
   1. Probes: Are these terms the same or different? What do they mean to you? A) personal doctor b) primary care doctor c) Generalist? D) family doctor? E) Specialist?
2. In our survey, we have to choose the best term for the place where such doctors see patients. What do you call the place where the <name given by patient> doctor sees patients?
   1. Probes: What of these terms: Clinic? Office? Hospital? Other?
3. How do you pay for your health care? Insurance? Out of pocket? Company pays?
4. What do you know about supplemental health insurance?
   1. Probe: Do you have any?

**Gender (Male/Female )Relations**

**I would like you to think back to a time when your doctor was a male (state female if the participant is a male)?**

1. What was it like having a doctor that is male (state female if male participant)? How does your experience with the doctor change when the doctor is female (or male if male participant)?
2. Are there times when it matters to you if the doctor is a male or female? Why?
3. Are there times when having a male or female doctor is not an concern?
4. Are there other qualities besides male/female that are important?
5. Are there any times you can share with me when having a female (male if male participant) was important to you?
6. What about when the doctor was a male (female if male participant)?
7. What information does your doctor need to know about the match of gender between the doctor and patient?
8. When your doctor is not of the same gender as you, what could make your experience better?

**Transition:** Many people rely not only on doctors, clinics and hospitals to take care of their health, but also on their religion, traditional healers or treatments, herbs, and food. I want to ask you about how you might have used these things to take care of your health.

**FOLK DOMAINS**

**1. Religious treatments**

1. How does your religion affect what you do about your health?

Probe: Can you give me some examples?

- 1. Are there any particular things your religion guides you to do to keep you healthy?
  2. Are there any particular things your religion guides you to do to get better when you get ill?

1. When you decide to see a doctor, how does your religion affect who you decide to see? (Clarification: By this I mean the things you do when you get ill or when you decide to go to a doctor or someone else for help with your illness?)
2. What information does your doctor need to know about your religion’s teachings and your health?
3. Can you tell me about anything that hospitals or doctors did differently for you because of your religious beliefs?

**2. Supplements/Vitamins (use word patient prefers)**

1. What kinds of supplements/vitamins do you take for your health besides medications?
2. What kinds of supplements/vitamins do you take when you are sick besides medications?
3. What information does your doctor need to know about these supplements/vitamins and your health?
4. Anything else?

3. **Herbal Medicine Treatments**

1. What kinds of treatments do you take for your health besides medications?
2. What kinds of treatments do you take when you are sick besides medications?
3. What information does your doctor need to know about these treatments and your health?
4. Anything else?

**3. Mind-body treatments (consider using the term ‘traditional body treatments’)**

1. When ill, sometimes people seek help from other kinds of healers that are not doctors. Can you tell me about what kinds of healers you have used? [Clarification, a Hakim or religious leaders are types of these other healers. What kind do you use?]

**b) Traditional body treatments describe treatments like acupuncture, cupping (hijama), cauterization (kaiy) or coin rubbing.**

**Probes:** Healing with the Quran (addressed under #1)? Bone setting, blood letting (phlebotomy).

- 1. Are there any traditional body treatments that you use to keep yourself healthy?
  2. Are there any traditional body treatments that you use when you get sick?
  3. Are there any other body treatments that you are using?

1. What does your doctor need to know about your use of these healers and traditional body treatments for your health?
2. Anything else?.

**4. Nutrition. Sometimes people take specific foods like honey or drinks like teas for their health?**

1. What kinds of foods do you use for your health?
   1. Anything else?
2. What kinds of drinks do you use for your health?
   1. Anything else?
3. What does your doctor need to know about your use of foods, drinks for your health?
   1. Anything else?

*Interviewer will ask related questions necessary for clarification.*

In our research, we are happy to share a copy of the results of our study.

If you would like a summary of our research, we would like to send a copy to you. If you have any questions or comments on these results, you will have a chance to let us know at that time.

Would you like a copy? Ask yes or no? If yes, then get information. If not, end the interview.

**Name**

**Address**

Thank you very much for participating in this research.
